# Calcium Enhances the Effectiveness of Melatonin in Improving Nutritional Properties of Soybean Sprouts and Germination Under Salt and Cadmium Stress

**DOI:** 10.3390/ijms26030878

**Published:** 2025-01-21

**Authors:** Arjun Adhikari, Mahesh Sapkota, Raddella Nishani Savidya, Ajayi Tolulope Tosin, Muchanji Adam, Mohammad Naushad Alam, Eun-Hae Kwon, Sang-Mo Kang, Shifa Shaffique, In-Jung Lee

**Affiliations:** Department of Applied Biosciences, Kyungpook National University, Daegu 41566, Republic of Korea; sapkota@knu.ac.kr (M.S.); nishanisrd@gmail.com (R.N.S.); ajayitolu84@knu.ac.kr (A.T.T.); adammuchanji@gmail.com (M.A.); alam97ad@gmail.com (M.N.A.); eunhae.kwon1@gmail.com (E.-H.K.); kmoya@hanmail.net (S.-M.K.); shifa.2021@knu.ac.kr (S.S.)

**Keywords:** abiotic stresses, antioxidants, mineral elements, nutrients, germination, sustainable agriculture, yield

## Abstract

Salinity and cadmium exposure to agrarian land lowers crop yield and imposes toxicity in the food chain, ultimately affecting sustainable agriculture. Melatonin (Mel) and calcium (Ca) have been reported as potent regulators of plant growth and stress resistance. Based on this scenario, this study investigated the sole and combined effects of Mel and Ca on improving the antioxidant properties, mineral content, germination of sprout, and stress tolerance of soybean seedlings under salt and cadmium (Cd) stress. Optimal doses of 20 µM Mel and 1 mM Ca were identified to enhance sprout quality and seed germination. Treatments with Mel > 20 µM inhibited germination, while the combination of Mel (20 µM) and Ca (1 mM) significantly improved germination, mineral content (Ca, P, K), and antioxidant properties, including DPPH(2,2-Diphenyl-1-picrylhydrazyl) activity, polyphenols, flavonoids, and superoxide dismutase (SOD) activity. However, melatonin > 50 µM could completely cease the sprouting, whereas a Ca concentration of up to 10 mM was observed to be normal in sprouting. Additionally, this combination reduced malondialdehyde (MDA) levels and enhanced the proline, indicating decreased oxidative stress in soybean seedlings under stress conditions. Among various treatments tested, the Mel-Ca combination was most effective in enhancing sprout biomass, antioxidant activity, and seed viability under Salt+Cd stress. These findings underscore the synergistic role of Ca in optimizing melatonin pretreatment for stress mitigation in soybean seeds and also address the precaution for a possible negative impact of melatonin effects.

## 1. Introduction

Abiotic stress and crop toxicity resulting from climate change and massive anthropogenic activities have imposed adverse challenges to a sustainable agro-food system [1]. With global food demand surging and over a billion more people expected by 2050, enhancing agriculture sustainability and nutritional value is vital to preserving ecosystem resilience and ensuring future agricultural productivity [2]. Failure to act will exacerbate food security challenges, deepening hunger and poverty, particularly in developing regions [3]. Microgreens and sprouts are becoming staples in modern diets, especially in high-density urban and rural areas. Their fast growth and rich nutritional value make them an ideal choice amidst declining arable land. Seed pretreatment with safe nutrient elements is an effective strategy to enhance the nutritional properties, quality, germination rates, vigor, and overall health of sprouts and microgreens [4,5]. It encompasses a range of methods applied before sowing to enhance seedling growth and resilience against pests, diseases, and environmental challenges [6]. In the case of soybean, a critical source of dietary protein and nutrients, pretreatment methods can also improve sprout quality for consumption and processing, offering nutritional, functional, and culinary benefits [7].

However, soybean cultivation faces significant threats from abiotic stresses such as salinity and heavy metal contamination, particularly cadmium (Cd) [8]. These environmental stressors disrupt essential physiological processes, including water uptake, nutrient assimilation, enzyme activity, and protein synthesis, ultimately reducing germination rates, impairing seedling establishment, and causing crop failure [9,10]. Salinization, a major issue in arid and semi-arid regions, and Cd pollution from industrial activities and excessive fertilizer use further exacerbate these challenges [11].

Plants naturally employ various biochemical and molecular strategies to counter these stresses, such as antioxidant production, ion homeostasis regulation, and stress-related hormone synthesis [12]. Among these, melatonin and calcium have emerged as pivotal regulators of plant stress tolerance and growth [13]. Melatonin, primarily recognized as a hormone in animals, exhibits significant benefits in plants, including antioxidant activity, stress resistance, and regulation of germination and sprout development [14]. Calcium, a vital nutrient in plants, plays a crucial role in cell wall stability, signaling, and metabolism [15]. When used as a pretreatment, calcium has been shown to enhance germination by modulating hormone activity and enzyme regulation while mitigating the toxic effects of heavy metals and salinity stress [16]. This dual role makes calcium a promising candidate for improving plant tolerance to abiotic stresses, including salinity and Cd toxicity [17].

The synergistic application of melatonin and calcium has shown potential in enhancing seedling growth and stress resilience [18]. Together, these two agents could form a powerful combination that helps plants tolerate various environmental stressors, including salinity and metal toxicity, by integrating antioxidant defense with structural fortification. Moreover, melatonin is a hormone essential for synchronizing circadian rhythms and regulating the onset and quality of sleep [19]. Consuming specific foods boosts melatonin levels and enhances plasma antioxidant status [20]. Food sources of melatonin have demonstrated health benefits, including enhanced antioxidant capacity and improved sleep duration [21,22]. One study suggested that dietary intake of kidney bean sprouts increased melatonin levels in the bloodstream in rats which was nearly equal to the treatment feed with synthetic melatonin [23]. Similar results were observed when rats were fed lentil sprouts, where plasma melatonin levels rose by 70%, reaching a maximum concentration of 45.4 pg/mL at 90 min, which was 1.2-fold higher than synthetic melatonin. These also improved the antioxidant capacity, suggesting their potential to boost melatonin naturally through diet [24]. Furthermore, walnuts containing melatonin (3.5 ng/g) significantly increased plasma melatonin levels and antioxidant activity in rats, highlighting their potential as a dietary source of melatonin [25,26,27]. Hence, it can be concluded that consuming melatonin-rich food products can significantly impact the levels of melatonin and related compounds in the body.

This study aims to investigate the combined effects of calcium and melatonin on soybean sprout quality, focusing on germination viability and stress resistance under salinity and Cd toxicity. The findings could provide valuable insights into integrated strategies for improving soybean resilience and productivity in stress-prone environments, advancing sustainable agricultural practices, and meanwhile, enhancing the nutrients in diet may impose a positive impact in aiding health benefits.

## 2. Results

### 2.1. Growth Parameters of Sprout

**Figure 1:**
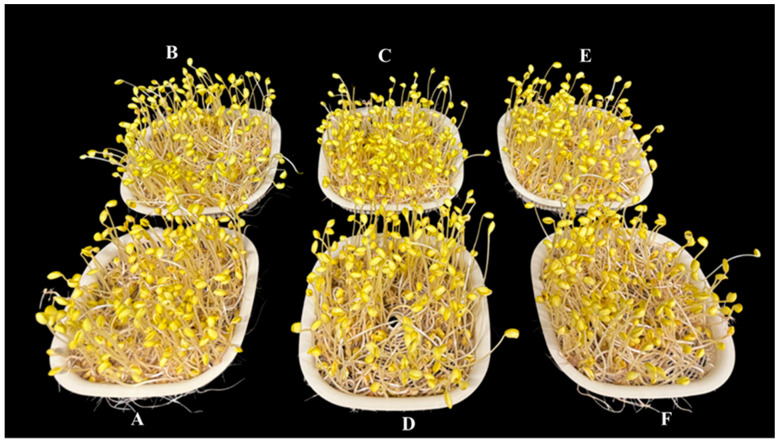
Visual observation of sprout growth on the 6th Day ((**A**); Control, (**B**); Ca 1 mM, (**C**); Mel 20, (**D**); CaMel, (**E**); MelCon, (**F**); CaCon).

- Biomass of sprout
  The total biomass of the sprout was considerably increased by 7% in Ca-Mel treated groups and by 5% with Ca 1 mM treated groups. Continuous Mel treatment reduced the fresh weight by 4%, whereas no significant differences were observed in fresh weight with treatment of melatonin alone when compared to the control; Table 1.
- Hypocotyl length of sprout
  The hypocotyl length was significantly increased with the treatment of Ca, Mel, and Ca-Mel by 9, 8, and 7%, respectively. The continuous treatment of melatonin (MelCon) reduced the hypocotyl growth by 12%, whereas continuous treatment of calcium (CaCon) increased the hypocotyl length by 5% when compared to the control; Figure 1 and Table 1.
- Radical length of Sprout
  The radical length was significantly increased by 13%, 7%, 22%, and 19% with the treatment of Ca, Mel, Ca-Mel, and CaCon, respectively, compared to the control. However, the continuous treatment of melatonin significantly reduced the radical length of the sprout by 12%; Table 1.

### 2.2. Antioxidant Properties of Sprout

**Figure 2:**
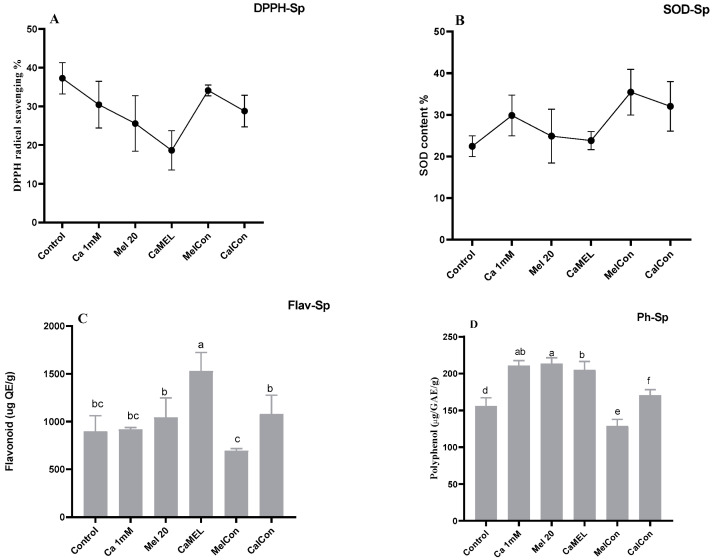
Antioxidant properties of soybean sprout (**A**) DPPH, (**B**) SOD, (**C**) flavonoid, (**D**) polyphenol. Sp represents sprouts. Error bars represent the mean ± standard deviation, with each data point calculated from at least three replicates. Different letters above error bar represent statistically significant differencesconducted using Duncan’s Multiple Range Test (DMRT) with a significance level of *p* < 0.01.

- SOD
  Superoxide dismutase (SODsp) activity in soybean sprouts increased under all treatments compared to the control (water only). Calcium (Ca 1 mM) enhanced activity by 24.80%, melatonin (Mel 20 µM) by 9.86%, and the combination of calcium and melatonin (CaMel) by 5.77%. Continuous melatonin treatment (MelCon) showed the highest increase of 36.72%, followed by continuous calcium treatment (CaCon) with a 29.98% increase. Continuous treatments proved more effective in enhancing SODsp activity; Figure 2.
- DPPH
  Here, a contrasting trend was observed in between melatonin alternate treatments vs. continuous treatment. DPPH content was reduced by 15% and increased by 12% with Mel 20 treatment and MelCon, respectively; Figure 2.
- Polyphenol
  The phenolic content showed a significant increase across all treatments, except for MelCon, which exhibited a 17% reduction. In contrast, the phenolic content increased by 26%, 27%, 33%, and 9% under the treatments of Ca, Mel, Ca-Mel, and CaCon, respectively; Figure 2.
- Flavonoid
  A similar trend was observed in the flavonoid content of the sprouts, with increases of 2%, 13%, 41%, and 16% under the treatments of Ca, Mel, Ca-Mel, and CaCon, respectively, when compared to the control. In contrast, the MelCon group showed a 22% reduction in flavonoid content; Figure 2.

### 2.3. Ca, P, and K Content of Sprout

**Figure 3:**
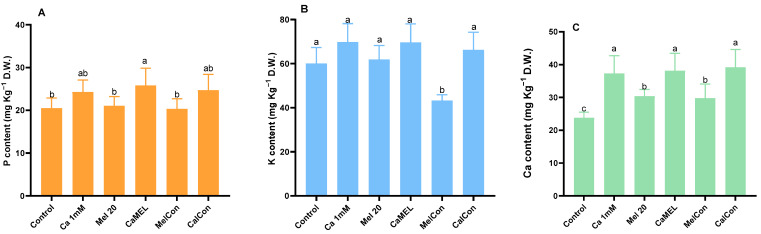
Quantification of Mineral nutrients in soybean sprouts after the treatment of Mel/Cal/MelCon/CaCon. (**A**) phosphorous (P), (**B**) potassium (K), (**C**) calcium (Ca). Error bars represent the mean ± standard deviation with a different letter representing statistically significant differences. Each data point represents the mean of at least three replicates based on Duncan’s Multiple Range Test (DMRT, *p* < 0.01).

Phosphorus (P), potassium (K), and calcium (Ca) contents varied across treatments compared to the control (water only). For P, the highest increase was observed with CaMel (20.55%), followed by CaCon (16.96%) and Ca 1 mM (15.48%), while Mel 20 µM showed a marginal increase (2.63%) and MelCon resulted in a slight decrease (−0.97%). For K, Ca 1 mM and CaMel both showed similar increases (13.87% and 13.71%, respectively), while CaCon increased it by 9.32%. Mel 20 µM caused a minor increase (2.86%), whereas MelCon significantly reduced K content (−28.09%). For Ca, all treatments enhanced content, with CaCon showing the highest increase (39.18%), followed by CaMel (37.58%), Ca 1 mM (36.15%), MelCon (24.89%), and Mel 10 µM (21.63%); Figure 3.

### 2.4. Soybean Seed Germination and Seedlings Resilience Under NaCl+Cd Stress

#### 2.4.1. Germination Rate

**Figure 4:**
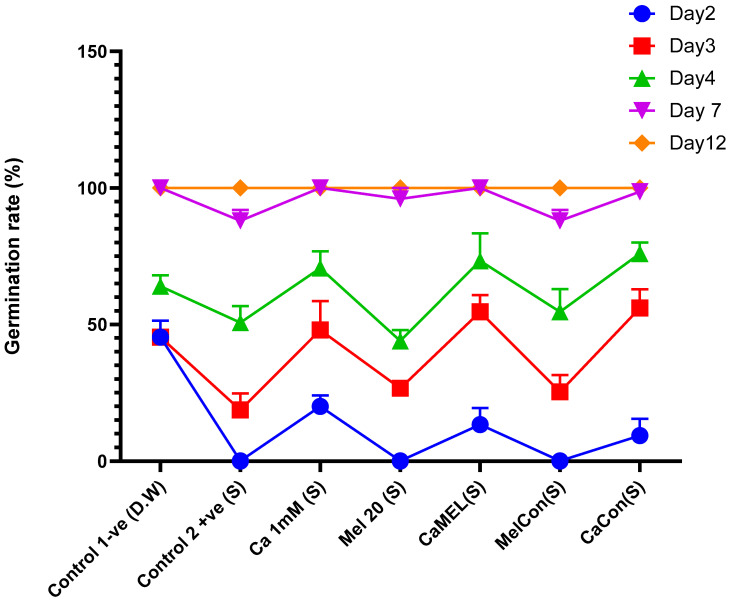
Germination percentage of soybean seedlings at different days after sowing. (S) represents Cd+NaCl stress. D.W. indicates distilled water.

**Figure 5:**
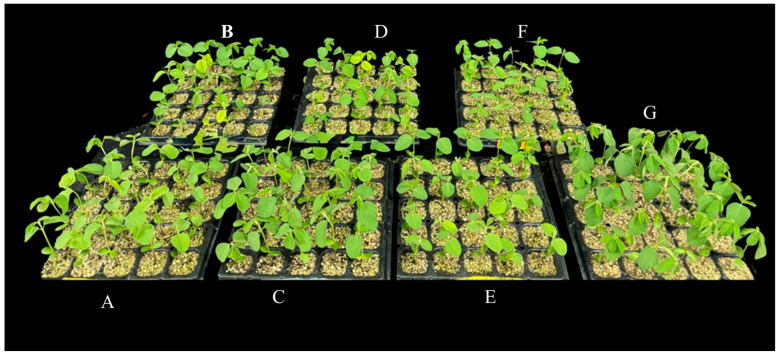
The visual observation of soybean seedling after 12 Days. ((**A**); Control 1 −ve, (**B**); Control 2 +ve, (**C**); Ca 1 mM, (**D**); Mel 20, (**E**); CaMel, (**F**); MelCon, (**G**); CaCon). Despite all the seeds attaining 100% germination by the end of 12 Days, the NaCl+Cd significantly delayed the seedlings emergence period, meanwhile lowering its vigor and strength. Cal-Mel treated seeds showed significant resistance against NaCl+Cd stress improving its morphological characteristics.

Seed germination percentages varied significantly by Day 7 across treatments. The negative control (D.W) and all treatments except the positive control (Cd+NaCl) achieved 100% germination by Day 12, making Day 7 the critical point for evaluating treatment effects. Calcium (Ca 1 mM) and the combination of calcium and melatonin (CaMel) showed strong early and intermediate germination effects, with CaMel achieving the highest rates by Day 7 (up to 84%). Continuous melatonin treatment (MelCon) exhibited slower initial germination but showed a noticeable improvement by Day 7, reaching up to 92%. Continuous calcium treatment (CaCon) demonstrated consistent germination progress, reaching 96–100% by Day 7. These results highlight the effectiveness of CaMel and CaCon in promoting faster germination, while MelCon, despite a slower start, provided significant intermediate improvement under stress conditions Figure 4 and Figure 5.

#### 2.4.2. Morphological Attributes

Seedling growth parameters (shoot length; SL, root length; RL, and biomass) showed significant variation across treatments under Cd+NaCl stress. The negative control (D.W) maintained the highest baseline values for all parameters, while the positive control (Cd+NaCl) exhibited sharp declines in SL (−45.4%), RL (−37.06%), and biomass (−16.52%). Among the treatments, CaMel showed the most balanced improvement, significantly enhancing SL (50.38%), RL (27.51%), and biomass (22.74%). Ca 1 mM increased SL (18.40%) and biomass (28.89%), but reduced RL (−69.45%). Mel 10 had moderate positive effects on RL (27.51%) and biomass (22.74%) with a slight increase in SL (5%). CaCon enhanced RL (45.91%) and biomass (17.95%) but had a smaller impact on SL (5%). MelCon improved RL (23.46%) but showed negative effects on SL (−3.91%) and a significant decrease in biomass (−81.86%). Overall, CaMel was the most effective treatment for improving seedling growth under stress conditions; Table 2.

#### 2.4.3. Chlorophyll Content and Soil pH

Soil pH showed slight variations between the negative and positive controls and across the treatments, with most values staying relatively stable. The chlorophyll content of plants was significantly affected by treatments. The negative control (D.W) exhibited the highest baseline chlorophyll content (303.87), while the positive control (Cd+NaCl) showed a drastic reduction (−47.07%), indicating stress-induced degradation. Among treatments, CaCon (46.93%) and Ca lmM (41.93%) were the most effective in enhancing chlorophyll content. Mel 20 and CaMel provided moderate improvements (29%), while MelCon showed only a negligible increase (0.88%); Table 2. These results suggest that calcium-based treatments are most effective in mitigating stress effects and improving chlorophyll retention.

#### 2.4.4. Antioxidant Like Activities of Soybean Seedlings Under Stressed Condition

**Figure 6:**
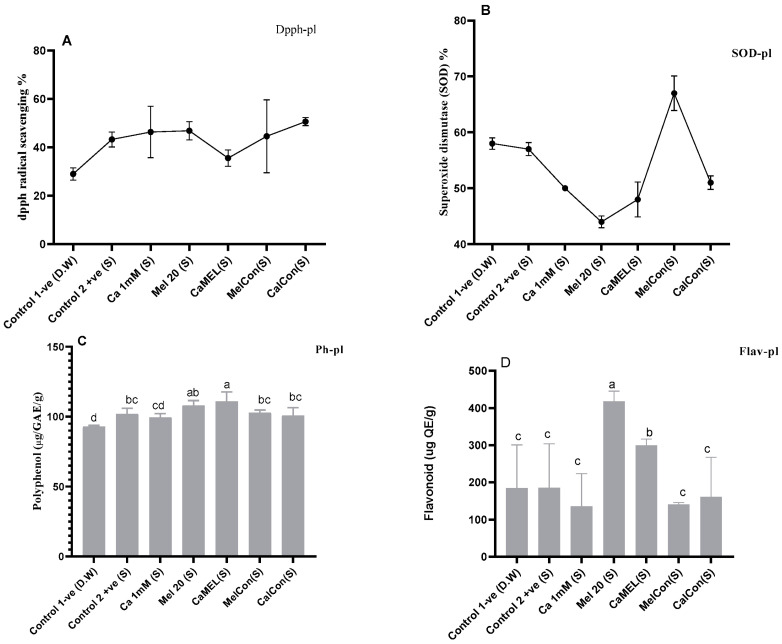
The antioxidant properties of soybean seedlings emerged on NaCl+Cd contaminated soil: (**A**) DPPH, (**B**) SOD, (**C**) polyphenol, and (**D**) flavonoid. (S); Salt+Cd stress. pl; plants. Error bars represent the mean ± standard deviation. Each data point represents the mean of at least three replicates. Bars with different letters are significantly different at *p* ≤ 0.01. D.W. indicates distilled water.

SOD activity decreased under most treatments compared to the negative control. The positive control showed a negligible change (−0.25%), while CaCon (−12.66%) and Ca 1mM (−14.56%) had moderate reductions. Mel 20 and CaMel exhibited the largest declines (−30.65%). In contrast, MelCon improved SOD activity (+14.55%), highlighting its potential for antioxidant enhancement; Figure 6.

Polyphenol content increased marginally under Mel 10 and CaMel (5.65%), while other treatments showed minimal or negative changes. CaCon and Ca 1mM had slight reductions (−1.10% and −2.51%, respectively). The positive control showed a moderate increase (9.87%); Figure 6.

“Flavonoid content was significantly enhanced by Mel 10 and CaMel (55.64%), with Ca 1 mM and MelCon showing declines (−36.44% and −32.05%, respectively). CaCon had a moderate reduction (−15.07%). The positive control showed negligible improvement (+0.26%), similar to the negative control; Figure 6.

Among treatments, CaCon and MelCon showed promising antioxidant effects by enhancing DPPH and SOD activity, while Mel 20 and CaMel significantly boosted polyphenols and flavonoids, indicating their potential to improve stress tolerance. However, reductions in SOD activity under most treatments warrant further investigation

#### 2.4.5. MDA and Proline Content of Soybean Seedlings Treated with Ca/Mel/Ca-Mel Under NaCl+Cd Stress

**Figure 7:**
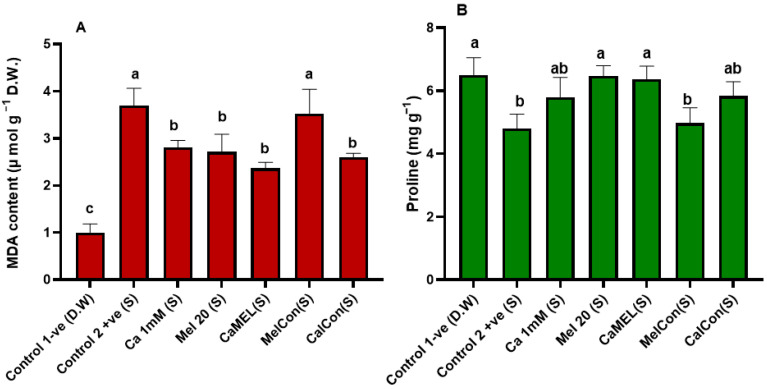
Quantification of (**A**) malondialdehyde (MDA) and (**B**) proline content of soybean seedlings grwon under NaCl+Cd stress. (S) represents Salt+Cd stress. D.W. indicates distilled water. Error bars represent the mean ± standard deviation. Each data point represents the mean of at least three replicates. Bars with different letters are significantly different at *p* ≤ 0.05.

Under Cd+NaCl stress, treatments significantly reduced MDA content, indicating a mitigation of oxidative damage. The positive control exhibited a sharp increase in MDA (+269.38%), while CaCon showed the greatest reduction (−42.13%), followed by Mel 20 and CaMel (−36.36% each). Proline content, a marker of stress tolerance, decreased slightly in the positive control (−10.77%). Mel 10 and CaMel showed the highest increases in proline content (+10.31%), highlighting their effectiveness in enhancing stress tolerance. Overall, CaCon was most effective in reducing oxidative stress, while Mel 20 and CaMel improved proline accumulation, enhancing stress resilience Figure 7.

## 3. Discussion

### 3.1. Soybean Seed Priming and Sprout Development

Soybean (*Glycine max*) is highly susceptible to abiotic stresses such as salinity and cadmium toxicity. The pretreatment of soybean seeds represents a vital agricultural practice with far-reaching implications for crop quality, resilience, and productivity [28]. In this study, the combined use of calcium (Ca) and melatonin (MT) as seed priming agents demonstrated significant potential in mitigating abiotic stresses such as salinity and cadmium (Cd) toxicity in soybean.

### 3.2. Sprout Quantity and Quality

The Ca/Mel/Ca-Mel treatment significantly enhanced the hypocotyl and radical length of the soybean sprout. On visual observation, the appearance color was light yellow which fits for consumers’ preference. The overall fresh weight of the sprout was significantly increased, where the Ca-Mel combination accounts for the highest amount. It was also observed that continuous melatonin treatment could significantly reduce the radical and hypocotyl growth, subsequently reducing the total biomass. These treated soybean sprouts were further tested for germination and resistance test under Cd+NaCl-contaminated soil.

### 3.3. Seed Germination and Seedlings Resilience

We first tested the germination with a 100 mM and 150 mM NaCl concentration. However, in these concentrations, the germination % was zero. Upon reducing the concentration to 50 mM NaCl, we observed that soybean seeds showed a considerable germination rate. The seeds treated with calcium and melatonin significantly improved germination, resulting in 100%, even under stress conditions such as high salinity or cadmium toxicity. The untreated seeds were also germinated; however, the germination time was delayed and the seedlings’ visual appearance and morphological attributes were adversely affected. When pretreated together, melatonin and calcium can effectively enhance the stress tolerance of soybean seeds more than with either treatment alone. These findings align with previous reports highlighting the role of Ca and MT in enhancing plant stress tolerance by improving physiological and biochemical traits [29,30]. The increased seed vigor and resilience observed in primed seeds suggest that melatonin and calcium treatments play an essential role in preparing seeds for challenging growing conditions, thus improving overall crop establishment and yield potential.

### 3.4. Impact on Nutrient Content and Antioxidant Properties in Sprout

The nutritional composition of soybean sprouts, including key elements such as potassium (K), calcium (Ca), and phosphorus (P), can be influenced by the application of calcium and melatonin. Supplementing with calcium can result in increased calcium content within plant tissues, contributing to better overall growth. When combined with melatonin, a potent antioxidant, this treatment could potentially enhance nutrient uptake, boosting the levels of not only calcium but also other essential minerals like potassium and phosphorus [31]. In our study, the Ca, P, and K content were significantly enriched by the treatment of Ca-Mel sprouts.

Further studies on antioxidant properties, including the evaluation of DPPH (2,2-diphenyl-1-picrylhydrazyl), polyphenol, flavonoid, and superoxide dismutase (SOD) content in soybean sprouts, indicate that calcium and melatonin treatments positively influence these compounds, which are linked to antioxidant activity and nutritional value. DPPH assays measure the free radical scavenging ability of plant extracts, and the treatment with calcium and melatonin has been shown to enhance this activity. Calcium can activate enzymes involved in polyphenol biosynthesis, while melatonin may increase the accumulation of polyphenols by regulating the expression of polyphenol-related genes or enzymes.

In addition, melatonin and calcium may influence the biosynthesis of flavonoids, another group of important antioxidants. Calcium ions are involved in key signaling pathways that regulate flavonoid production, and melatonin’s regulatory effects on flavonoid biosynthesis could further enhance the flavonoid content in treated soybean sprouts. This could lead to sprouts with improved antioxidant properties, contributing to health-promoting effects when consumed [32,33].

Superoxide dismutase (SOD), an essential antioxidant enzyme, catalyzes the dismutation of superoxide radicals into oxygen and hydrogen peroxide, providing critical protection against oxidative stress [34]. Calcium is known to activate various enzymes, including SOD, and its presence in seed priming treatments could enhance SOD activity, thereby improving the plant’s ability to combat oxidative damage. Overall, the combination of calcium and melatonin priming is likely to result in increased radical scavenging activity, enhanced polyphenol and flavonoid content, and increased SOD activity, all of which contribute to the improved antioxidant properties and nutritional value of soybean sprouts.

### 3.5. Reduced Oxidative Damage in Seedlings Subjected to NaCl+Cd

Excessive Cd and Na accumulation impairs plant growth via oxidative injury. Malondialdehyde (MDA) is a commonly used marker for oxidative stress in plants, and its levels increase significantly when plants are subjected to environmental stresses like salinity or heavy metal toxicity [12]. In this study, priming soybean seeds with calcium and melatonin resulted in reduced MDA levels, indicating that the treatment helped alleviate oxidative damage caused by stress. Our results are in line with Munir et al. [35], who demonstrated that the melatonin treatment reduced Cd accumulation and enhanced SOD, POD, CAT, and APx, and reduced cellular oxidative damage in rice [35]. Similar results were reported in faba bean [36], *B. napus* [37], tobacco [38], and Dracocephalum kotschyi genotypes [39], which showed the improvement of Ca^2+^, K^+^, chlorophyll, proline (Pro), and the CAT, POD, and SOD in plants treated with Ca^2+^/Melatonin under Cd and NaCl stress. This reduction in MDA content suggests that the antioxidant properties of melatonin, combined with the stabilizing effects of calcium, help protect the seeds from oxidative stress, ensuring healthier seedlings and more robust plant growth under challenging conditions.

In the case of seed germination, Li et al. [40] demonstrated that the melatonin counteracts ABA by reducing Ca^2+^ efflux and H_2_O_2_ accumulation to induce germination through ABA catabolism and GA_3_ biosynthesis. It was reported that the priming of dry seeds with melatonin improves lignification, maintains osmolyte homeostasis, and ensures balanced mineral uptake, mitigating the adverse effects of Cd stress on buckwheat seedlings [41]. It was also demonstrated that melatonin inhibited Cd translocation, on cotton seedling growth [42], restricting Ni accumulation on fenugreek [43], promoted cucumber seed germination and root growth under chilling stress [44], and improved soybean morphological attributes under drought and salt stress [45]. It has been reported that proline plays a crucial role in regulating ABA synthesis, stomatal conductance, and stress resistance [12]. The current findings showed that the Ca-Mel treatment significantly elevated proline content, which might have played a role in ABA catabolism, preventing Cd translocation, balancing ionic fluxes, and sustaining the metabolism to ensure germination.

### 3.6. Precaution for Melatonin and Calcium Application for Enhancing Sprout Quality and Seedling Resilience Under Salt+Cd Stress

In the current study, seed germination above NaCl 100 mM was completely ceased despite the treatment of Ca/Mel/Ca-Mel treatment. Similarly, the continuous sprinkle of 20 µM melatonin may also account for lower sprout growth and yield. These seeds may have a low viability of germination in both normal and stressed conditions. However, there is no negative effect of calcium up to a 10 mM concentration. There was a slight decrease in length and yield of sprout from the 9–10 mM Ca treatment, which probably indicates that above these concentrations may result in an inhibitory effect. There are only a few reports that showed the negative effect of melatonin application in seed germination. Our findings corroborate to some extent with a few of the researchers, such as Lv, et al. [46] who demonstrated that low melatonin concentrations (10–100 µM) had no effect, but higher levels (500–1000 µM) significantly inhibited germination of Arabidopsis. The possible mechanism reported by the author was that the ABA and melatonin synergistically suppressed germination, while GA and auxin counteracted this effect. Mutations in melatonin biosynthesis genes (SNAT or ASMT) promoted germination, whereas ASMT overexpression inhibited it [46]. These mechanisms have been highlighted by several authors on their research where it is reported that the abiotic stresses impair seed germination (SG) by deteriorating seed quality, reducing germination potential, and seed vigor. Melatonin (MEL) enhances SG under stress by regulating ionic homeostasis, storage protein hydrolysis (salinity), C-repeat binding factor signaling (cold), starch metabolism (heat, heavy metals), aquaporins, and osmolyte accumulation (drought). Common MEL-mediated responses include gibberellin biosynthesis, abscisic acid catabolism, redox homeostasis, and Ca^2+^ signaling, supporting sustainable crop yields under stress [47,48]. Further study based on these findings in our research may add new insights in the future.

### 3.7. Notable Insights on Melatonin in Diet, Animal, and Plant Interactions Highlighting the Current Research Significance

The significance of melatonin in the human body is that it is widely reported that melatonin positively influences the immune system by boosting cytokine production, proving beneficial in viral and bacterial infections and cancer treatment [27]. It was observed that the use of melatonin could completely remove pesticide residues of chlorothalonil (10 mM) and malathion (1 mM) from contaminated grains and improved antioxidants and fatty acids in sprouts [49]. Based on these findings, we can predict that the Ca-Mel combination may be more useful in ensuring the safety and quality of the grains.

Long-term storage leads to seed deterioration, reducing nutrient and antioxidant levels. Melatonin treatment enhanced the antioxidant content of aged seeds, producing sprouts with increased total phenolics, improved ferric-reducing power, and higher DPPH radical scavenging capacity. These findings highlight melatonin’s potential to convert aged seed reserves into antioxidant nutrients, providing a valuable alternative use for deteriorated seeds in food production [50].

The effectiveness of seed pretreatment, however, is contingent upon various factors such as the type of seed, species, and intended growing conditions. While pretreatment with melatonin and calcium can significantly improve seed performance, improper application methods or excessive dosages may lead to negative consequences, either compromising seed viability or causing unintended environmental impacts. Thus, optimizing the conditions and methods for seed pretreatment is essential to harness the full potential of these treatments.

## 4. Materials and Methods

### 4.1. Experiment to Improve the Quality and Sprouting of Soybean

#### 4.1.1. Screening of Optimum Doses of Ca and Melatonin for Sprouting of Soybean

To determine the optimal treatment concentrations, a range of concentrations for each element was applied during the screening phase. Calcium (Ca) treatments included 1 mM, 5 mM, 15 mM, 30 mM, and 50 mM solutions, with distilled water serving as the control. For melatonin treatments, several amounts of concentration were tested in the range of 10 µM–1 mM using a Zaigle sprouting machine (Zaigle Co., Ltd., Heojun-ro, Gangseo-gu, Seoul, Republic of Korea).

#### 4.1.2. Plant Material and Sprouting Procedure

A total of 500 uniform soybean seeds were used for the initial screening to identify the optimal concentrations of calcium and melatonin for sprout development. The seeds were germinated using a Zaigle sprouting machine, which administered water for one minute every hour. On the first day, distilled water was used for irrigation, followed by alternating days of chemical treatment and water application Table 3.

**Sprout Cultivation Treatments:**
  ○ **Control (water only):** Seeds were treated with distilled water (D.W.) only.
  ○ **Ca 1 mM:** Seeds were treated with 1 mM calcium.
  ○ **Mel 20:** Seeds were treated with 20 µM melatonin.
  ○ **CaMel:** Seeds were treated with a combination of 1 mM calcium and 20 µM melatonin.
  ○ **MelCon:** Continuous treatment with 20 µM melatonin.
  ○ **CaCon:** Continuous treatment with 1 mM calcium.

#### 4.1.3. Measurement of Growth Parameters

After six days of germination, the root length, shoot length, and biomass of the sprouts were measured. The optimal concentrations identified for the growth parameters were 1 mM for calcium, 15 µM for iron, and 20 µM of melatonin for a 24 h treatment Details explained in Appendix B.

#### 4.1.4. Preparation of Seeds for Seedlings Experiment

Soybean seeds were subjected to the specified treatments during the sprout germination phase. After 3 days of treatment, the seeds were transplanted into Cd+NaCl-contaminated soil to evaluate their germination success and resistance during the seedling stage. Following one cycle of water and chemical treatment, 25 seedlings from each treatment group were transferred to trays for the subsequent NaCl and Cd contaminated soil experiment.

### 4.2. Effect of Mel/Ca Treated Seeds Sown Under NaCl+Cd Contaminated Soil

#### 4.2.1. Study Site and Experimental Setup

The experiment was conducted in a laboratory and polyvinyl house at Kyungpook National University, located in Daegu, South Korea (35.53° N, 128.36° E). The temperature within the polyvinyl house was controlled at 30 ± 6 °C, with natural daylight and a relative humidity ranging from 60% to 70%. Soybean seeds of the Pungsanamul variety, South Korea, were utilized in this study. The seeds were germinated using a Zaigle sprouting machine (Zaigle Co., Ltd., Heojun-ro, Gangseo-gu, Seoul, Republic of Korea. For the NaCl+Cd stress experiment, the seeds were planted in 5 × 10 polypropylene seed trays equipped with germination holes. A growth medium consisting of a 1:4 mixture of horticultural soil and sandy soil was used in the trays. NaCl equivalent to 50 mM and CdSO_4_, a dose that is ecologically toxic as reported by Tóth, et al. [51], i.e., (40.908 g NaCl + 280 mg Cd in 28 kg soil), was mixed in 28 Kg soil.

#### 4.2.2. Experimental Treatments

The experimental design incorporates specific treatments to evaluate the effects of calcium (Ca), melatonin (Mel), and their combinations on soybean sprouting and seedling resistance under abiotic stress conditions (Cd+NaCl contamination). The seedlings’ treatments are now categorized as two controls, namely, Control 1 (−ve): seeds were treated with distilled water only and Control 2 (+ve, S): seeds were exposed to Cd+NaCl stress without additional treatments. The remaining treatments are exactly the same as that of sprout cultivation. Other biochemical analysis parameters are detailed in the Appendix A section.

#### 4.2.3. Observation and Sample Collection

Germination and growth were monitored throughout the experiment. Field samples were collected 18 days after transplanting (DAT), immediately stored in liquid nitrogen, and subsequently kept at −80 °C for preservation. For the sprouting experiment, six-day-old sprouts were harvested, and their root length, shoot length, and biomass were measured before being stored at −80 °C. These samples were then freeze-dried for further analysis.

### 4.3. Analysis of Antioxidant Activities

- **DPPH Radical Scavenging Activity Determination**
  DPPH radical scavenging activity was assessed using a freshly prepared 0.05% DPPH solution in absolute methanol as described by Wang et al. [52]. Equal volumes (100 µL each) of DPPH solution and sample extract were mixed in microplates. The reaction mixture was incubated in the dark for 30 min at room temperature (22–25 °C). For the control, 100 µL of DPPH solution was combined with 100 µL of methanol. After incubation, the absorbance of the reaction mixtures was measured at 517 nm using a microplate reader (Multiskan GO, Thermo Fisher Scientific, Vantaa, Finland). The DPPH radical scavenging activity was calculated using the following formula:
  DPPHradical scavenging activity (%) = [1 − ((A − Ao)/(B − Bo))] × 100
- **Quantification of Total polyphenol and Flavonoid Content**
  Total polyphenol and flavonoid content was quantified using a modified colorimetric method [53]. For flavonoids, 30 µL of 5% NaNO_2_ was added to 30 µL of sample extract, followed by 60 µL of 10% AlCl_3_ after 5 min of incubation. After vortexing, the mixture was incubated for another 5 min, and then 200 µL of 1 M NaOH was added. Absorbance at 500 nm was measured using a microplate reader (Multiskan GO, Thermo Fisher Scientific, Vantaa, Finland). Results were expressed as quercetin equivalents (QE) in mg/g extract.
  For polyphenols, 50 µL of extract was mixed with 1 mL of 2% sodium carbonate solution, followed by 50 µL of 1N Folin–Ciocalteu reagent. After incubating for 30 min in the dark, absorbance at 750 nm was measured using a microplate reader (Multiskan GO, Thermo Fisher Scientific, Vantaa, Finland). Results were expressed as gallic acid equivalents (GAE) per gram of sample.
- **Superoxide Dismutase (SOD)-like Activity**
  The SOD-like activity was analyzed using a method described by Ha et al. [54]. Frozen plant shoot samples were ground using a grinder, and a reaction mixture consisting of 300 µL of 50 mM Tris-HCl buffer (pH 8.5) + 10 mM EDTA, 200 µL of 7.2 mM pyrogallol, and 200 µL of sample extract was incubated at 25 °C for 10 min. After completion of the reaction, 50 µL of 1N HCl was added to stop the reaction. The absorbance of the oxidized pyrogallol was measured at 420 nm using a microplate spectrophotometer (Multiskan GO, Thermo Fisher Scientific, Vantaa, Finland).

### 4.4. Analysis of the Extent of the Lipid Peroxidation and Proline Content

MDA, a byproduct of lipid peroxidation commonly used as a marker of oxidative stress, was quantified using thiobarbituric acid-reactive substances [55]. Briefly, 0.5 g of fresh leaf tissue was extracted with 10 mL of 5% trichloroacetic acid, and the supernatant was collected. The solution was then mixed with thiobarbituric acid and incubated in a water bath at 85 °C for 25 min. After incubation, the sample was immediately cooled on ice to 4 °C. The extract was filtered, and the absorbance was measured at 600 nm and 532 nm using a spectrophotometer (Multiskan GO, Thermo Fisher Scientific, Finland).

The extraction and quantification of proline were performed following the method described by Shahzad et al. [56]. Whole plant samples (100 mg), finely ground, were hydrolyzed under vacuum using 6N HCl at 110 °C and subsequently at 80 °C for 24 h. The hydrolyzed residue was then dried, homogenized in 0.02N HCl, and filtered through a 0.45 μm membrane. The amino acids were quantified using an automatic amino acid analyzer (Hitachi, L-8900, Tokyo, Japan). The experiment was conducted in triplicate, and proline concentrations were determined by comparison with specific standard solutions.

### 4.5. Quantification of Mineral Elements (K, Ca, P)

Mineral elements (K, Ca, and P) were quantified using the protocol described by Adhikari et al. [57]. In this procedure, 0.5 g of freeze-dried sample was digested with 70% HNO_3_ on a heating plate at 110 °C for 1.5 h. To enhance the digestion process, H_2_O_2_ was added, and the mixture was processed using an Ultrawave microwave digestion system (Milestone, Shelton, CT, USA). The resulting supernatant was separated, filtered, and prepared for analysis. A 3 mL aliquot of the extract was diluted with deionized water to a final volume of 30 mL. The prepared solutions were analyzed for elemental composition using inductively coupled plasma mass spectrometry (ICP-MS; Optima 7900DV, PerkinElmer, Shelton, CT, USA) to ensure accurate quantification.

### 4.6. Statistical Analysis

The results were presented graphically using GraphPad Prism software (version 8.0.2). Statistical analyses were performed using SAS software (version 9.4). Duncan’s Multiple Range Test (DMRT) was employed to compare means, with a statistical significance set at *p* < 0.01, *p* < 0.05. The experiment followed a completely randomized design (CRD). For the measurement of morphological parameters of sprout hypocotyls and radicles, 20 samples were obtained from each treatment replicate. For seed germination, 25 seedlings per treatment were considered. For all other parameters, data points were based on at least three to six replicates.

## Figures and Tables

**Table 1:** Effect of Ca and Mel treatment on morphological attributes of soybean sprout.

Trt	Radical (Cm)	Hypocotyl (Cm)	Total Biomass (g)
Control	12.71 ± 0.90 b	11.89 ± 0.32 b	198.64 ± 6.88 b
Ca 1 mM	11.81 ± 0.66 a	13.02 ± 0.51 a	209.33 ± 9.01 a
Mel 20	8.83 ± 0.81 a	12.94 ± 0.35 a	198.64 ± 6.88 b
Ca-Mel	5.72 ± 0.28 a	12.77 ± 0.64 a	209.33 ± 9.01 a
MelCon	5.65 ± 0.34 c	10.4 ± 0.29 c	188.72 ± 7.15 c
CaCon	10.62 ± 0.36 a	12.5 ± 0.70 a	198.64 ± 6.88 b

Each data point represents the mean of at least twenty replicates (mean ± standard deviation; *n* = 4) for radical and hypocotyl, whereas at least three replicates were measured for total biomass measurement. Columns with different letters are significantly different at *p* ≤ 0.01.

**Table 2:** Effect of Mel/Ca/Mel-Ca treated soybean sprouts on morphological characteristics of seedlings grown under NaCl+Cd contaminated soil.

Trt	Shoot Length (cm)	Root Length (cm)	Biomass (g)	Soil pH	Chlorophyll (SPAD)
Control 1−ve (D.W)	8.13 ± 0.70 a	11.30 ± 0.78 ab	1.32 ± 0.28 ab	6.83 ± 0.01 a	303.87 ± 57.22
Control 2 +ve (S)	4.43 ± 1.05 c	7.11 ± 1.53 bc	1.10 ± 0.23 bc	6.61 ± 0.01 b	160.84 ± 24.61
Ca 1 mM (S)	5.43 ± 0.92 c	4.20 ± 6.14 c	1.54 ± 0.33 c	6.63 ± 0.01 b	276.98 ± 75.04
Mel 20 (S)	4.66 ± 0.75 c	9.81 ± 0.99 ab	1.42 ± 0.17 ab	6.61 ± 0.09 b	227.58 ± 104.74
CaMEL (S)	6.66 ± 1.52 c	12.52 ± 1.33 a	1.91 ± 0.23 a	6.50 ± 0.01 b	222.72 ± 89.88
MelCon (S)	4.26 ± 1.04 b	9.29 ± 2.00 ab	0.60 ± 0.10 ab	6.70 ± 0.01 b	162.27 ± 20.27
CaCon (S)	4.66 ± 2.02 c	13.15 ± 0.86 a	1.34 ± 0.28 a	6.63 ± 0.01 b	293.07 ± 147.97

Each data point represents the Hmean of at least six replicates represented by mean ± standard deviation. Columns with different letters are significantly different at *p* ≤ 0.01. (S) represents Cd+NaCl stress.

**Table 3:** Treatment procedure for sprouting fortification with different elements.

Day	Control (D.W)	Ca 1 mM	Mel20	CaMel	MelCon	CaCon
1	D.W.	D.W.	D.W.	D.W.	Treatment	Treatment
2	Treatment	Treatment	Treatment	Treatment	Treatment	Treatment
3	D.W.	D.W.	D.W.	D.W.	Treatment	Treatment
4	Treatment	Treatment	Treatment	Treatment	Treatment	Treatment
5	D.W.	D.W.	D.W.	D.W.	Treatment	Treatment
6	Treatment	Treatment	Treatment	Treatment	Treatment	Treatment

Note: The treatment procedure was followed as an alternating cycle between one day of water treatment and one day of chemical treatment, whereas MelCon and CaCon were continuously treated without cycle of water. D.W. indicates distilled water.

## Data Availability

The data will be made available upon request.

## Appendix A. Experimental Parameters and Treatments for Soybean Research

1. **Parameters Assessed**
  The experiment focuses on assessing various biochemical, physiological, and morphological parameters in soybean sprouts and seedlings. These are categorized as follows:

- **Sprout-Specific Parameters:**
  ○ **DPPHsp:** Antioxidant activity (2,2-diphenyl-1-picrylhydrazyl assay).
  ○ **SODsp:** Superoxide dismutase activity.
  ○ **Phenolsp:** Total phenolic content.
  ○ **Flavsp:** Flavonoid content in sprouts.
  ○ **Nutritional Elements:** Phosphorus (P), potassium (K), calcium (Ca).
  ○ **Growth Parameters:** Radical length, hypocotyl length, and fresh weight (Frwt).
- **Seedling and Soil-Specific Parameters:**
  ○ **Growth Parameters:** Shoot length (Sl) and root length (Rl).
  ○ **Biomass**: Total biomass accumulation.
  ○ **Stress Markers:** Malondialdehyde (MDA) and proline (Prol) content.
  ○ **Antioxidant and Polyphenol Assays:** DPPHpl, SODpl, polyphenol content (Phpl), and flavonoid content (Flavpl).
  ○ **Soil pH:** Changes in soil pH.

## Appendix B

Calcium (Ca) treatments up to 10 mM demonstrated a positive impact on growth, whereas melatonin (Mel) concentrations exceeding 50 µM exhibited toxic effects. Sodium chloride (NaCl) concentrations greater than 100 mM completely inhibited seed germination, regardless of Ca or Mel supplementation. Melatonin at 20 µM could exert a positive impact; however, its continuous application, such as prolonged soaking of seeds, should be avoided. Instead, an alternate cycle of changing every 12 h with normal or distilled water is recommended to produce healthy and stress-resilient sprouts. Cadmium (Cd) exposure delayed germination and weakened seedling vigor; however, it did not significantly inhibit the overall germination process.
